# To Abort a Cold

**Published:** 1899-09

**Authors:** 


To Abort a Cold.—Max Nassauer asserts that an incipient cold in the head can be checked every time if the nose is thoroughly rinsed out with a weak solution of potassium permanganate, which seems to have a specific action upon the germs causing the trouble. He claims that the public will have a higher respect for the profession when it is proved that colds can be successfully aborted by following the physician's directions. He checks colds in the first hour or so, and thus escapes all the catarrhal and bronchial annoyance that follows in their train. He has a strong solution of potassium permanganate on hand, about what can be taken up on the tip of a small knife, to half a liter water. A few drops of this strong solution are added to warm water until it is colored a pale pink. After blowing the nose vigorously, both nostrils are rinsed out well with this weak solution, allowing the fluid to run out through the other nostril and through the mouth. Each nostril is then wiped out with cotton on the finger to mechanically remove all remaining germs. A small dry plug of cotton is then pushed well up into each nostril and the nostrils filled with the weak solution, with the head held back, allowing the cotton to soak it up. The cotton is left undisturbed for about an hour, for the warmth and moisture to produce their effects, when the plugs are expelled by blowing the nose. Even an established cold is favorable influenced by this treatment, but it is most effective when the sneezing, tickling and increased secretions announce the advent of the cold, which he considers a highly contagious infection.

